# Evaluation of Clinical and Immunological Responses: A 2-Year Follow-Up Study in Children with Allergic Rhinitis due to House Dust Mite

**DOI:** 10.1155/2013/345217

**Published:** 2013-04-27

**Authors:** Heleen Moed, Roy Gerth van Wijk, Rudi W. Hendriks, J. C. van der Wouden

**Affiliations:** ^1^Department of General Practice, Erasmus MC-University Medical Center Rotterdam, P.O. Box 2040, 3000 CA Rotterdam, The Netherlands; ^2^Department of Allergology, Erasmus MC-University Medical Center Rotterdam, P.O. Box 2040, 3000 CA Rotterdam, The Netherlands; ^3^Department of Pulmonary Medicine, Erasmus MC-University Medical Center Rotterdam, P.O. Box 2040, 3000 CA Rotterdam, The Netherlands; ^4^Department of General Practice, VU University Medical Center, 1081 HZ, P.O. Box 7057, 1007 MB Amsterdam, The Netherlands

## Abstract

*Background*. Allergic rhinitis is a disease with polarization towards Th_2_ and a defect of regulatory T cells. Immunological changes have been reported after immunotherapy treatment. However, there is not much known about the natural course of allergic rhinitis with respect to clinical manifestation and the relation with immunological responses. *Objective*. To evaluate clinical symptoms of allergic rhinitis, in relation to *in vivo* allergen-specific skin responses and *in vitro* allergen-specific effector and regulatory T cells determined at baseline and after two years. *Methods*. From a large trial, 59 children were randomly selected. The following variables were compared: clinical symptoms, allergen skin tests, specific IgE, T-cell proliferation, IL-5, IL-13, IFN-gamma, IL-10, TGF-beta, CD4^+^CD25^hi^ cells, and Foxp3 expression. *Results*. Allergic symptoms had decreased after two years. Whereas skin test reactions correlated between years 0 and 2, there was no change in the size of the reaction. Also, proinflammatory reactions did not change after two years, with a positive correlation between years 0 and 2. No relevant changes were observed with respect to regulatory cells. *Conclusion*. Whereas, comparable to immunotherapy, allergic complaints decrease, the immunological changes of specific T-cell activity (both effector cells and regulator cells) which are observed after immunotherapy, do not change.

## 1. Introduction

Allergic rhinitis (AR) is a common chronic illness affecting 10% to 20% of the children worldwide [1], and its prevalence has significantly increased among children over the last two decades. AR is clinically defined as a symptomatic disorder of the nose induced by an IgE-mediated inflammation after exposure to an allergen. In the immediate response to an antigen (the early-phase allergic reaction), histamine and other inflammatory mediators are released from mast cells in the nasal mucosa [2]. This causes the characteristic nasal symptoms which include sneezing, itching, rhinorrhoea, and nasal congestion. A late-phase allergic reaction occurs approximately 4 to 12 hours after antigen exposure, with nasal congestion as the predominant symptom. During this phase, inflammatory cells such as eosinophils and T cells infiltrate the mucosa. The Th_2_-polarized immune response especially, with secretion of cytokines such as IL-4, IL-5 and IL-13 and a defect of Th1 cells producing IFN-gamma, play a central role in the late phase allergic response [2–4].

It has been observed that the immune response to allergens is the result of a fine balance between allergen-specific Th_2_ cells and T regulatory cells (Tregs) [5]. Tregs include a broad spectrum of CD4^+^ T-cell subpopulations such as natural thymic-derived Treg cells (nTreg) characterized by their CD4^+^CD25^+^ phenotype expressing high levels of Foxp3 [6] and inducible Tr_1_ cells (iTreg) which suppress immune function by the secretion of IL10 and TGF-beta [7–9].

From the recent literature it becomes clear that it may be possible to interfere with the natural course of AR by specific immunotherapy (SIT) [9–13]. SIT may induce changes that skew Th_2_- to Th_1_-type responses [14] and by means of tolerance induction. One of the key mechanisms behind this tolerance induction includes induction of Treg-type cytokines (IL-10 and TGF-beta) [9, 10] and increased frequencies of CD4^+^CD25^+^ cells [11].

There is an unexplained variability in the clinical course of allergic rhinitis—with persistence in some individuals and either progression or remission in others. There is a lack of longitudinal investigations examining the natural history of AR. In the present study, we investigate the natural course of AR regarding clinical manifestations of the disorder and immunological responses in a followup of 2 years in children with house dust mite allergy. We evaluate clinical symptoms, *in vivo* skin responses due to allergen and *in vitro* allergen-specific effector T cells and Treg cells during the natural course of the disease.

## 2. Methods

### 2.1. Study Design

This study is a part of the randomized placebo-controlled trial ISRCTN91141483 which evaluates sublingual immunotherapy with low-dose house dust mite allergen (2.03 mcg/mL or 700 BU/mL Der p1) in children with AR [15, 16]. Patients entered the study either in September–December 2005 or in September–December 2006 for a period of approximately two years. Written informed consent was obtained. The study was approved by the Ethical Review Board of Erasmus MC-University Medical Center Rotterdam. 

As described elsewhere, [16] neither this low-dose form of SLIT was effective with regard to the reduction of allergy complaints, asthma complaints, intake of rescue medication and disease-specific quality of life, nor was there any difference in reported side effects (both local and general) between SLIT or placebo treatment, indicating that the dosis of the investigated product was a low to mediate reaction. Analysis of placebo (*n* = 30) and verum (*n* = 29) groups separately in children participating in the present study revealed neither differences between verum and placebo regarding clinical symptoms (*P* value varied between 0.06 and 0.86) nor *in vivo* skin responses (*P* value between 0.17 and 0.80) or *in vitro* measurements of pro-inflammatory markers (*P* value between 0.16 and 0.85) or regulatory T cells (*P* value between 0.10 and 0.89). Therefore, we decided to merge the two groups and consider them as one group which received placebo during two years. This merged group forms the basis for the present study.

### 2.2. Patient Selection

From the main trial, fifty-nine children were randomly selected and invited to participate in this elaborate study. As in the main study, children (aged 6–18 years) with AR and established HDM allergy were selected from the electronic medical records in general practice. The inclusion criteria were presence of specific IgE antibodies to HDM in serum (≥0.7 kU/L), a history of allergic rhinitis during at least 1 year and a nasal symptom score of at least 4 out of 12 (see below). Before scoring symptoms, nasal corticosteroids were withheld for 4 weeks before the study period. During the total study period patients were allowed to use rescue medication (provided by us, i.e., levocetirizine tablets, xylometazoline nasal spray, and levocabastine eyedrops) or another allergy or asthma medication as long as they wrote it down on their diary cards (see below). The presence of asthma was assessed using the International Study of Asthma and Allergies in Childhood (ISAAC) core questionnaire [17].

### 2.3. Measurement of Nasal, Eye, or Asthma Symptoms

All participants or their parents scored their nasal, eye, and asthma (lung) symptoms on diary cards at baseline (1 month in October or in November) and after two years (3 months in September–December). Nasal symptoms (sneezing, itching nose, watery running nose, and nasal blockage), eye symptoms (itching, tearing, and redness), and asthma symptoms (wheeze/breathless and dry cough during night) were scored on a 0–3 scale (0 = none, 1 = mild, 2 = moderate, and 3 = severe). In total, a maximal daily cumulative nasal symptom score of 12, eye symptom score of 9, and lung symptom score of 6 could thus be obtained. 

A mean symptom score was determined by calculating the mean daily score over the entire diary period (i.e., one month at baseline and three months after 2 years). Only diaries with at least 50% of the filled-out pages were included in the analyses. In case patients used additional medication for their allergy or asthma, they were asked to document their use in the patient diary throughout the 2-year period.

### 2.4. Skin Testing

Allergy skin testing was performed at baseline and after 2 years by intracutaneous injection of 0.02 mL *Dermatophagoides pteronyssinus* in the forearm (concentration 30 SQ U/mL, manufactured by ALK-Abelló, Nieuwegein, The Netherlands). We chose to perform an intracutaneous skin test rather than the usual skin prick test because intracutaneous injection of the allergen is the most feasible and convenient way to induce a late-phase response after the early-phase skin response [18]. As a positive control, histamine (concentration of 0.01 mg/mL) was injected, and the negative control was dilution buffer. Reactions were read after 15 min (early response) and after 6 h (late response). The area of the skin response in mm^2^ was measured by a specially developed scanning programme. The early-phase response was expressed as a histamine equivalent intra-cutaneous index or HEIC index. The late-phase response was expressed as the area of the skin response in mm^2^. Children were not allowed to take antihistamines within 24 h before skin testing.

### 2.5. Detection of House Dust Mite-Specific IgE

Serum IgE antibodies to *D. pteronyssinus* were determined using the CAP-RAST system (Pharmacia, Uppsala, Sweden), according to the manufacturers instructions.

### 2.6. House-Dust-Mite-Specific T-Cell Proliferation

Blood was drawn at baseline and after 2 years before skin testing. Peripheral blood mononuclear cells (PBMCs) were isolated from heparinized blood by density centrifugation on Ficoll-Paque Plus (GE Healthcare, Uppsala, Sweden). PBMCs were used in a lymphocyte proliferation test (LPT). Cells were resuspended in complete medium (RPMI + HEPES + glutamax supplemented with gentamicin (Gibco, Gibco BRL, Life Technologies, Rockville, MD, USA) and 5% heat-inactivated human serum (Sanquin, Rotterdam, The Netherlands) and stimulated by culturing in the presence or absence of 2 IR/mL *D. pteronyssinus* (Stallergènes, France). All cell cultures were performed in quadruplicate in a final volume of 200 mL with a cell concentration of 2 × 10^6^ cells/mL in 96-well round-bottomed microtitre plates. To determine HDM-specific proliferative responses, cells were cultured for 5 days at 37°C, 5% CO_2_, and 95% humidity. During the last 16 h, cells were pulsed with 0.5 *μ*Ci/well ^3^H thymidine (Pharmacia, UK). Radioactivity was measured with a *β*-plate reader, and the proliferative capacity was assessed by the stimulation index (SI), calculated as the ratio of mean ^3^H thymidine uptake in stimulated to that in non-stimulated cultures. The SI was considered positive when it exceeded 2.0.

### 2.7. House-Dust-Mite-Specific Cytokine Production by ELISA

To determine HDM-specific cytokine production, 1 mL of PBMCs with a concentration of 2 × 10^6^ cells/mL was cultured in duplicate in a 24-well plate with or without 2 IR/mL *D. pteronyssinus* (Stallergènes France). After 5 days of culture, supernatants were harvested and stored at −20°C until testing. IL-5, IL-13, IFN-*γ*, IL10, and TGF-beta cytokine production was measured following the manufacturer's instructions (eBioscience, San Diego, CA, USA or R&D Systems, Abingdon, UK). HDM-induced cytokine production was assessed by subtracting the cytokine concentration of nonstimulated from that of stimulated culture supernatants.

### 2.8. Detection of CD4^+^CD25^hi^ Cells by FACS

For phenotypical analysis, cells were washed with FACS buffer (0.05% NaN_3_, 2% BSA in PBS) and stained for 30 min on ice protected from light. For the detection of CD4^+^CD25^hi^ T cells the following anti-human antibodies were used: FITC-conjugated anti-hCD3 (UCHT1, eBioscience), PE-conjugated anti-hCD25 (M-A251, BD) APC-conjugated anti-hCD4 (RPA-T4, eBioscience). Aspecific binding was prevented by the use of 2% Heat Inactivated (HI) human AB serum during staining. Afterwards the cells were washed three times with FACS buffer and measured. Data acquisition was performed by flow cytometry (FACSCalibur; BD Biosciences) and data analysis was performed using FlowJo software (Treestar, Coata Mesa, CA)).

### 2.9. Measurement of Foxp3 Levels by Means of Real-Time Quantitative PCR

Quantitative RT-PCR for hFoxP3 was performed on RNA isolated from HDM-stimulated PBMC's (as described above). Total RNA was isolated with RNeasy Mini Kit (Qiagen) and treated with DNAseI, according to manufacturer's protocol. 100 ng RNA was used as a template for cDNA synthesis, using Superscript II reverse transcriptase (Invitrogen) and random hexamer primers. Quantitative PCR was performed with Taqman Universal PCR Mastermix (Applied Biosystems), preformulated primers (hFoxP3 and housekeeping gene hHPRT) and probe mixes (“Assay on Demand”, Applied Biosystems). PCR conditions were 2 min at 50°C, 10 min at 95°C, followed by 40 cycles of 15 s at 95°C and 60°C for 1 min using an ABI PRISM 7300 (Applied Biosystems). PCR amplification of the housekeeping gene was performed during each run for each sample to allow normalization between samples.

### 2.10. Statistical Analysis

Values are presented as mean (with standard deviation, SD), median (with interquartile range, IQR), and median estimated difference between baseline (year 0) and year 2. Comparisons were performed by nonparametric analysis, using Wilcoxon signed rank test for comparisons between baseline data and data after 2 years. The Spearman rank test (*R*_*s*_) was used to assess the correlations between year 0 and year 2. A *P* value of 0.05 was considered statistically significant. All analyses were performed using statistical software SPSS (version 18).

## 3. Results

The baseline characteristics of the 59 children included in the study are described in an earlier study [19]. Of the 59 children, 42 (71.8%) completed the 2-year study period. The most important values are summarized in Table 1. Mean and median values (with SD or IQR) of clinical symptoms, *in vivo* allergen-induced skin tests, and *in vitro* specific pro-inflammatory and regulatory responses are displayed in Table 2.

Clinical symptoms of the nose, eye and lung all decreased significantly two years after baseline measurement.  Table 3 shows a median paired estimated difference of −1.34 (*P* < 0.0001) for nose symptoms, −0.29 (*P* = 0.02) for eye symptoms and −0.33 (*P* < 0.0001) for lung symptoms. There was a significant positive correlation between values of year 0 and year 2 for all clinical symptoms (*R*_*s*_ between 0.56 and 0.61; *P* < 0.0001).

Reactions of *in vivo* HDM specific skin tests (both early as well as late skin reactions) did not significantly change when measured two years after the baseline measurement (Table 3, *P* = 0.09 or *P* = 0.07, resp., Table 3). Whereas there were no significant changes in time, skin responses showed a positive correlation when skin responses of year 0 and year 2 were compared (*R*_*s*_ = 0.44; *P* = 0.005 for the early skin response or *R*_*s*_ = 0.5; *P* = 0.001 for the late skin response).

Relevant proinflammatory variables for AR (i.e., HDM specific IgE, T-cell proliferation, IL-5, IL-13, and IFN-gamma) were comparable between year 0 and year 2 (Table 3, *P* > 0.05 except for IFN-gamma which showed a significant decrease). Whereas values did not significantly increase or decrease, there was a significant correlation between data from year 0 and year 2 for all these markers (*R*_*s*_ between 0.35 and 0.86), except for IL-5 (*R*_*s*_ = 0.27).

The frequency of regulatory CD4^+^CD25^hi^ cells had increased two years after baseline measurement. However, other markers characteristic for regulatory cells (Foxp3, IL-10, TGF-beta) did not increase in time (in fact for IL-10 a decrease could be detected, Table 3). Correlations between year 0 and year 2 for these variables were nonsignificant (except for IL-10).

**Figure 1:**
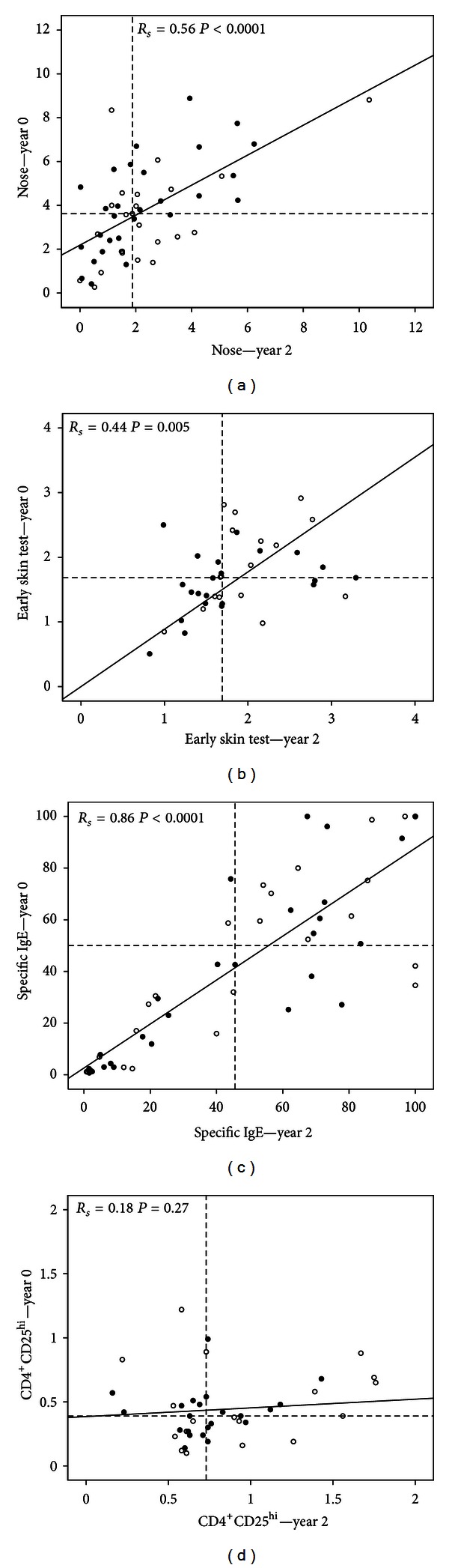
Correlation between clinical and immunological values determined at baseline and two years later. (a) patient rated nose symptoms (*n* = 52), (b) response of early skin test (*n* = 42), (c) specific IgE (*n* = 53), and (d) regulatory CD4^+^CD25^hi^ T cells (*n* = 41). Open bullets: verum SLIT-treatment during two years, closed bullets: placebo treatment during two years (see Section 2), dashed lines: median values, and *R*_*s*_: Spearman Rank test.

Figure 1 summarizes the above-described findings with a decrease of clinical symptoms after 2 years in combination with a positive correlation between data of year 0 and year 2 (represented by nasal symptoms in Figure 1(a)), comparable skin-test values for year 0 and year 2, with a positive correlation between the two years (early skin test is presented in Figure 1(b)), no significant change in proinflammatory reactions (in Figure 1(c) represented as spec IgE) with a high correlation between measurements during the two years, and an increase of CD4^+^CD25^hi^ Treg cells without a correlation between the two years of measurement (Figure 1(d)). As can be seen in this figure, there is no difference in any of the values between patients receiving placebo treatment or verum-SLIT treatment (which they received during a period of two years in the original trial).

## 4. Discussion

Results of the present study show clinical symptoms rated by allergic patients at baseline and two years later in combination with immunological parameters measured *in vivo* or *in vitro*. In a time period of two years, clinical symptoms of the nose, eye, and lung significantly decrease in children with house-dust-mite-induced AR. Although children rate their allergy complaints as being less than at the beginning of the study, this decrease is not reflected in a decrease of one of the immunological parameters. 

A possible explanation for the reduction of symptoms is that allergic rhinitis decreases in time. Whereas there are many studies determining symptoms during followup of SLIT treatment, there is little information on the possibility of spontaneous remission of allergic rhinitis [12, 20]. Some studies have reported the relationship between age and improvement or decline of rhinitis over the years [21–23]. During an 8-year follow-up of patients with allergic rhinitis,  Nihlén et al. [21] found a 20% remission rate, which was higher in older patients. Broder et al. [22] described that remission with allergic rhinitis was more likely when the duration of the disease was shorter than 5 years.

Another plausible explanation for the reduction of symptoms might be that, due to participation in a study, patients are more aware of their allergy and take more care of their complaints, which may result in a reduction of their symptoms. In this study, patients took (placebo) medication during a period of two years. The effect of placebo treatment is substantial in patients participating in a study evaluating SLIT.

Whereas from this study it becomes clear that complaints of patients with AR decrease over time, it is demonstrated in a birth cohort study that the prevalence of patients developing AR increases over the time [24]. Therefore, when children get older the change of developing AR is increasing, but for the patients with complaints of AR the change of having comparable complaints two years later is decreasing.

Skin responses (measured directly after intradermal allergen injection and a late response with induction of inflammatory cells) remain constant over time. Whereas these responses are comparable at baseline and after two years, these data show a positive correlation, which means that values which are high at baseline are also high after two years, and low values remain low during the two years. It therefore appears that sensitization persists even in the cases with remission of symptoms. This is underlined by a study of Bodtger and Linneberg [25] who describe that in adult patients with house dust mite allergy, only 5.5% of the patients show a remission of specific IgE after eight years, whereas remission of symptoms occurred in 32.4% of the patients. Also, a recent study of Kong et al. [26] describes that five years after skin testing 96% remain skin test positive.

AR is characterized by high levels of allergen-specific IgE and secretion of the Th_2_ cytokines IL-4, IL-5 and IL-13 [3, 27, 28]. In this study, we do not see a decrease of these inflammatory markers two years after the baseline measurement, whereas patients rate their symptoms as better than before. In contrast to our findings, in patients who do not receive immunotherapy, allergen-specific T-cell responses can be reduced by means of specific immunotherapy (SIT). The literature shows that SIT is able to reduce allergic complaints in combination with a reduction of allergen-induced T-cell proliferation [14] and a shift in the Th2/Th1 cytokine balance, favouring type-1 T cells [29]. This shift could be a result of either decreased IL-4 production after allergen stimulation [14] or enhanced IFN-*γ* expression [30]. Some studies have found a reduction of allergen-specific IgE in combination with an increase of specific IgG levels due to SIT [30–32]. In the large randomized study [16], of which this present study is a component, we determined IgG1 and IgG4 levels in 163 children at year 0 and year 2. These results show that IgG levels remain constant over the time with a very high correlation between year 0 and year 2 (*R* is 0.74 for IgG and 0.83 for IgG4), which emphasises that the dose of allergen administrated might have been insufficient to induce tolerance.

Different studies demonstrate the effect of immunotherapy on regulatory mechanisms [9–12]. They describe a significant increase of IL-10, and TGF-*β* in patients undergoing SIT compared to untreated atopic patients (or patients receiving placebo). Moreover, the number of CD4^+^CD25^+^ cells expressing the transcription factor Foxp3 was increased after allergen stimulation in the immunotherapy group [13]. In our study, results related to regulatory T cells are conflicting. Whereas we see an increase of CD4^+^CD25^+^ cells, there is no correlation between data of year 0 and data of year 2. Moreover, other markers regarding regulation of the immune response (i.e., Foxp3, IL-10 and TGF-beta) do not increase. In fact, IL-10 decreases in time. From this, it is unlikely that regulatory cells play a role in this present study; self-rated allergy symptoms do decrease, but this is not the result of an increase of regulatory cells. However, we have to keep in mind that the number of patients used to answer this question is small, and we should be conservative with respect to this statement.

A limitation of the present study is that only 42 out of 59 children (71%) wanted to participate two years later. Some of these children were lost to followup (a total of seven children), and others still participated in the main SLIT study but did not want to do additional skin testing or venapuncture (a total of ten children). However, the total number of 42 children is still an acceptable number to study possible immunological mechanisms in relation to symptoms in a period of two years.

From this study, it can be concluded that whereas, comparable to SIT, allergic complaints decrease, the immunological changes of HDM-specific T-cell activity (both effector cells and regulator cells) which are observed after immunotherapy do not change. This decrease in clinical symptoms might be explained by the placebo effect, since patients participated in a study and took (placebo) medication during a period of two years. It may be concluded that SIT plays an active role in tolerance induction in the patients immune system; whereas during the two years of this study, allergic complaints reduce without any difference in immunological parameters as determined in this study. 

## Figures and Tables

**Table 1:** Baseline characteristics of the study population.

	*N* = 59
Age, years (mean, SD)	11.6 (3.0)
Gender, male (*n*, %)	28 (47.5%)
Wheeze/dyspnea last year (*n*, %)	37 (62.7%)
Multisensitization (*n*, %)	49 (83.1%)

**Table 2:** Mean (SD), median (IQR), and number of different variables for year 0 and year 2.

	Year 0					Year 2				
	Mean	SD	Median	IQR	*N*	Mean	SD	Median	IQR	*N*
Clinical symptoms										
Nose (score from 0–12)	3.8	2.2	3.62	3.46	59	2.3	1.95	1.84	2.06	52
Eye (score from 0–9)	1.15	1.32	0.67	1.39	59	0.65	1.1	0.21	0.79	52
Lung (score from 0–6)	0.87	1.13	0.37	1.33	59	0.36	0.9	0.028	0.15	52
HDM-specific skin test										
Early skin test (HEIC)	1.72	0.61	1.69	0.76	52	1.96	0.78	1.7	0.87	42
Late skin test (mm^2^)	871.4	771.6	759.2	1389.8	59	1112.4	904.6	1044.5	1418.28	42
HDM-specific proinflammatory response										
Specific IgE (kU/L)	43.23	34.78	38.1	62.45	59	47.58	34.81	45.6	62.3	53
Proliferation (SI)	6.84	6.18	4.78	5.52	59	5.59	6.73	3.29	5.22	41
IL-5 (pg/mL)	892.93	1963.66	329.66	793.15	59	384.4	549.35	194.82	515.00	41
IL-13 (pg/mL)	848.54	1243.31	313.05	1206.3	59	875.31	783.25	1183.44	1494.64	41
IFN-gamma (pg/mL)	97.81	123.61	55.67	139.94	59	58.39	101.85	26.93	72.73	41
HDM-specific regulatory response										
CD4^+^CD25^hi^ (% of CD3^+^)	0.44	0.26	0.39	0.32	59	0.88	0.46	0.73	0.44	41
FoxP3 (relative expression)	3.22	2.41	2.39	2.47	59	2.8	1.75	2.4	2.65	41
IL-10 (pg/mL)	81.75	157.33	21.32	95.02	59	21.81	39.0	9.24	24.7	41
TGF-beta (pg/mL)	105.13	258.26	0.00	27.27	59	221.35	383.86	58.72	305.75	41

SD: standard deviation.

IQR: Inter Quartile Range.

**Table 3:** Paired estimated difference between year 2, and 0 (median, CIs and *P*-value) and Spearman correlation.

	Paired estimated difference				Correlation	
	Median	95% CI*		Sign.^#^	*R*_*s*_^†^	Sign.
Clinical symptoms						
Nose (score from 0–12)	−1.34	−1.86	−0.85	<0.0001	0.56	<0.0001
Eye (score from 0–9)	−0.29	−0.62	−0.05	0.02	0.56	<0.0001
Lung (score from 0–6)	−0.33	−0.58	−0.13	<0.0001	0.61	<0.0001
HDM-specific skin test						
Early skin test (HEIC)	0.16	−0.03	0.35	0.09	0.44	0.005
Late skin test (mm^2^)	199.85	−12.55	458.2	0.07	0.5	0.001
HDM-specific pro-inflammatory response						
Specific IgE (kU/L)	1.95	−0.79	6.45	0.17	0.86	<0.0001
Proliferation (SI)	−0.67	−2.02	0.89	0.39	0.38	0.015
IL-5 (pg/mL)	−132.1	−450.62	69.51	0.19	0.27	0.087
IL-13 (pg/mL)	133.92	−210.18	538.48	0.46	0.36	0.022
IFN-gamma (pg/mL)	−44.84	−82.33	−4.00	0.01	0.35	0.027
HDM-specific regulatory response						
CD4^+^CD25^hi^ (% of CD3^+^)	0.44	0.3	0.56	<0.0001	0.18	0.27
FoxP3 (relative expression)	−0.12	−0.67	0.41	0.64	0.24	0.13
IL-10 (pg/mL)	−39.34	−67.66	−10.56	0.002	0.357	0.024
TGF-beta (pg/mL)	41.43	0.00	144.01	0.18	0.032	0.84

*CI: confidence interval Hodges Lehman.

^#^ *P*-value, Wilcoxon.

*R*_*s*_^†^: Spearman rank correlation between year 0 and year 2.
